# Notes and News

**Published:** 1906-01-20

**Authors:** 


### Notes and News.

Mr. A. W. Mayo Robson has been appointed to represent the Royal College of Surgeons of England at the fifteenth International Medical Congress to be held in Lisbon next April.

Mr. James Wilcock, of Blackburn, has commemorated his sixtieth birthday by giving £1,000 to the Blackburn and East Lancashire Infirmary, and £500 each to five other charities in Blackburn.

The accounts for the past year of the Hospital Saturday Fund closed on January 8. The net receipts amounted to £25,777, as compared with £24,232 collected in 1904, thus showing the substantial increase of £1,545.

The Kingston Board of Guardians have decided to apply to the Local Government to include phthisis in the list of notifiable diseases. They are also sending copies of their resolution to each Board of Guardians and Urban Council in the country requesting their co-operation.

A prize has been offered by the National Association for the Study of Epilepsy for the best essay on the etiology of that disease. The value of the prize is $300, and medical men of any nationality may compete. Intending candidates should apply for details of the competition to Dr. W. P. Spratling, Craig Colony for Epileptics, Sonyea, Livingston County, New York.

An interesting ceremony took place at St. Thomas's Hospital on Tuesday last, when a portrait of the Treasurer, Mr. J. G. Wainwright, was presented to the Hospital by the Lord Mayor, acting on behalf of the governing body. The portrait, which is by Mr. J. H. F. Bacon, A.R.A., was received by the senior almoner, Mr. Ambrose Boyson, and a replica was presented to Mrs. Wainwright. Mr. Wainwright has been a governor of St. Thomas's Hospital for forty years, and has always shown himself to be deeply interested in its welfare. He was appointed Treasurer fifteen years ago.

Mr. David Allan, of Aberdeen, who died in September last, in addition to other charitable legacies, bequeathed £1,000 to the Aberdeen Royal Infirmary.

Under the will of the late Mr. Yerkes a portion of the estate, estimated at £160,000, will, on the death of the widow, be devoted to the establishment of a hospital in America, and there will also be a large sum for maintenance.

Macclesfield General Infirmary benefits to the extent of £1,500 under the will of Mrs. Phœbe Elizabeth Lovatt, and Stockport Infirmary to the extent of £1,000 under that of Mrs. Jane Anne Pearson, both of whom were local ladies.

An endowment of £10,000 has been handed over to the Frank James Memorial Hospital at Cowes, of which Princess Henry of Battenberg is president, by Messrs. James, who founded the hospital. A brass tablet will be affixed to each bed stating that it was endowed in memory of Queen Victoria.

The late Sir John Burdon-Sanderson, Regius Professor of Medicine in the University of Oxford, has bequeathed £2,000 as an endowment for the laboratory of the pathological department of the University of Oxford, to provide for pathological research. The fund is to be vested in the professors for the time being of human anatomy, physiology, and pathology, who will have absolute discretion as to the application of the fund.

A meeting of the Assistants of the Society of Apothecaries will be held on January 23, at 8 p.m., in the Court Room of the Society's Hall, Water Lane, Blackfriars, E.C., to discuss the advisability of forming an association. Ladies and gentlemen holding the assistants' certificate are invited to attend. Those unable to be present, but in agreement with the suggested association, should communicate their views to Mr. Albert Howell, Hackney Union Dispensary, Rosebery Place, Dalston, N.E.

Mr. T. Barclay Cockerton, District Auditor of the Local Government, has recently had under his consideration the objections of the British Medical Association and other ratepayers to certain payments made by the London County Council to Mr. John Troutbeck, one of the coroners of the County of London. The payments in question were made to the coroner to reimburse him for fees paid to Dr. Freyberger in respect of post-mortem examinations performed by direction of the coroner. After hearing the arguments of the two parties in the disagreement and reviewing the whole facts of the case, Mr. Cockerton has come to the conclusion that he is bound to allow the whole of the payments. While arriving at this decision he desires to express his sympathy with those members of the medical profession who are affected by Mr. Troutbeck's general mode of procedure, and he proposes to refer to the matter in his report to the Council at the close of the audit.

